# *In vitro* antimicrobial and cytotoxic activity of Neem and Kirata herbal formulation mediated Silver nanoparticles

**DOI:** 10.6026/973206300181069

**Published:** 2022-11-30

**Authors:** Rebekah R, Saravana Dinesh SP, Nivethigaa B, Rajeshkumar S

**Affiliations:** 1 Department of Orthodontics and Dentofacial Orthopaedics, Saveetha Dental College and Hospitals, Saveetha Institute of Medical and Technical sciences(SIMATS), Saveetha University, Chennai - 77; 2 Nanobiomedicine Lab, Department of Pharmacology, Saveetha Dental College and Hospitals, Saveetha Institute of Medical and Technical Science, Saveetha University, Chennai -77, India

**Keywords:** Nanoparticles, AgNp, neem extract, kirata, green, green synthesis, antimicrobial property, cytotoxicity

## Abstract

Silver nanoparticles (AgNPs) gain great interest among noble metal nanoparticles due to their broad applications in medicine, dentistry, drug delivery, tissue and tumour imaging, biolabeling, and biosensing. The antibacterial, antifungal, antiviral, and antiparasitic activity of AgNPs is well documented in the literature. This study aimed to determine the antimicrobial and cytotoxic activity of Neem and Kirata herbal formulation-mediated silver nanoparticles against oral biofilm. The green synthesis of Neem and Kirata herbal formulation-mediated silver nanoparticles was done. The antimicrobial action against the strains of Candida albicans, Staphylococcus aureus, Enterococcus faecalis, and Streptococcus mutans were assessed. The results showed that the newly formulated nanoparticle had effective anti-microbial properties and decreased cytotoxic properties which make it advantageous for clinical applications and treatment modalities. It showed great potential for the nanoparticle in decreasing the bacterial effects and cytotoxic nature thereby providing future scope for clinical application in preventing oral biofilm formation and its deleterious effects.

## Background:

 Research and development of applied science at the atomic or molecular level is referred to as nanotechnology or nanoscience [1]. Nanotechnology has an impact on practically every aspect of modern life, including s ecurity and medicine. The development of nano dentistry, which is similar to nanomedicine, enables almost perfect oral health through the use of nanomaterials and biotechnologies [2]. Silver nanoparticles (AgNPs) are the most favoured noble metal nanoparticles due to their numerous uses in medicine, dentistry, drug administration, tissue and tumour imaging, biolabelling, biosensing and many other fields. The literature is replete with AgNP's antibacterial, antifungal, antiviral and antiparasitic properties [3,4]. The interaction of neutral plant extracts and nanoparticles is beneficial since it lowers the compound's toxicity and enhances its availability and ease of manufacture. It is common practice to use plants or isolated products of them as anti-inflammatory and antioxidant agents [3]. Azadirachta indica, commonly known as neem, has a wide range of medicinal properties. Chemicals such as azadirachtin, nimbinena, nimbin, nimbandial, diacetyl nimbinase and salanin are known to be present in neem extracts from the seeds, stems, flowers, and leaves which belong to the terpenoid group. The phenolic category also includes quercetin, rutin, and gallic acid, among others. The antibacterial, antiviral, antiprotozoal, insecticidal, antifungal and antioxidant effects have all been demonstrated for these chemicals [4]. Swertia chirata, commonly known as Chirayata in India, is a laxative and appetizer that has a healthy bitter taste and also has strong anti-microbial and anti-inflammatory properties [5]. The primary goal of the current study is to investigate Neem and Swertia chirata's in vitro effects by evaluating their antimicrobial effects. The phytochemical analysis shows the existence of the substances that support the plants' antibacterial activity [6]. Therefore, this study aimed to ascertain the antimicrobial activity against oral biofilm and the cytotoxic activity of silver nanoparticles herbally formulated from neem and kirata.

## Materials and Methods:

## Plant extracts formulation:

The powdered Kirata and Neem plant extracts were purchased and combined in equal parts. Neem and kirata powders totalling 2 g were weighed and taken separately. The extract powder was now dissolved in the conical flasks with the addition of 100 mL of distilled water. On a heating mantle, the mixture was heated to 60° Celsius. The extract was heated before being filtered with filter paper. The purified extract was collected and refrigerated at 4°Celsius.

## Nanoparticle synthesis:

The treatment of Neem and Kirata extract was done with 0.016 g of silver nitrate and 90mL of distilled water. The treated extract was then positioned in an orbital shaker at 120 rpm overnight. The synthesis of nanoparticles was noted on an hourly basis with the help of a double-beam UV spectrophotometer in the wavelength range of 250-650nm.

## Silver nanoparticle preparation:

The synthesized nanoparticle solution was centrifuged at 8000rpm for a period of 10 mins. After this process, the supernatant was discarded and the pellet was stored.

## Antimicrobial activity:

It was examined whether the corresponding nanoparticles had any antimicrobial action against the strains of Candida albicans, Staphylococcus aureus, Enterococcus faecalis, and Streptococcus mutans. For this experiment, the inhibitory zone was identified using Muller Hinton agar (MHA). The preparation and sterilization of MHA were done at 121 ° Celsius for 15 minutes at 15 lbs. Sterilized plates were filled with media and left to stand so that they could solidify. The well cutter was used to cut the wells, and test organisms were swabbed. The plates were filled with nanoparticles of varying concentrations and incubated at a temperature of 37 ° Celsius for 24 hours. After the incubation period, the inhibition zone was assessed.

## Cytotoxic activity:

Brine shrimp lethality assay:

Saltwater preparation:

200 mL of distilled water and 2g of non-iodized salt were weighed and dissolved. 6 to 8 mL of saline water was added to 6 well ELISA plates. To that, 10 nauplii were added and along with those different concentrations of nanoparticles ranging from 5 µL, 10 µL, 20 µL, 40 µL, and 80 µL were added. 24-hour incubation was performed on the plates. The ELISA plates were examined after 24 hours to count the living nauplii that were present. Using this data, the dead nauplii count was calculated using the following formula:

1 number of live nauplii/number of dead nauplii + the number of living nauplii *1

## Results:

### Antimicrobial activity:

The synthesized Neem and kirata herbal formulation mediated silver nanoparticles were evaluated for their antimicrobial activity by detecting the zone of inhibition against Streptococcus mutans Staphylococcus aureus, E. faecalis, and C. Albicans as shown in Figure 6. Strong antimicrobial activity was observed against the pathogens. The zone of inhibition increased along with an increase in the nanoparticle concentration. At 25 µL, 50 µL and 100 µL concentrations, the zone of inhibition was found to be highest against S. aureus. Amongst the bacterial pathogens, the antimicrobial activity was highest at 100 µL concentration. C. Albicans showed lesser effect compared to other bacterial microorganisms and the activity of the newly formulated nanoparticle was similar in all three concentrations.

### Plant extracts formulation:

Cytotoxic activity:

Cytotoxicity of the newly formed nanoparticles was tested by analyzing the death and inhibition of growth of the organisms under increasing concentrations. The cytotoxic activity was minimal in general. Only at 20, 40 and 80 µL concentrations, brine shrimp lethality was seen. Thus, only in higher concentrations, the slightly cytotoxic activity of the formulated nanoparticle was seen.

## Discussion:

Silver nanoparticles showed significant antibacterial activity against the selected Gram-negative foodborne pathogens. Thus, AgNPs might be a good alternative to develop as an antibacterial agent against multidrug-resistant strains of bacteria. The applications of AgNPs may lead to valuable findings in various fields such as medical devices and antimicrobial systems. The present study evaluated the effects of Neem and Kirata plant extracts based on their antimicrobial and cytotoxic properties. Strong antimicrobial activity was observed with the newly formulated nanoparticle and was found to be highest against S. aureus at the maximum concentration. The cytotoxic activity was also found to be comparatively decreased. Previous studies have also stated strong antimicrobial and less cytotoxic activity for Neem and Kirata plants individually. Susanti et al. stated that green synthesis of plant extracts and metal nanoparticles proved to have increased antimicrobial activity [7]. Ye et al. stated that the noble metal component eliminates the bacteria and cell membrane biofilm [8]. Loo et al also proved that silver nanoparticles showed high antibacterial activity against gram negative bacteria [9]. Silver nanoparticles synthesized chemically have lowest minimum inhibitory concentration with a value of 0.0063 mg/mL against E. coli [10]. Singh et al also observed inhibitory effect of biosynthesized silver nanoparticles against bacterial pathogens. Azadirachta indica or the Neem plant is recognised for a lot of its beneficial properties, especially in the field of medicine [11]. Kangasanthosh et al. 2015 stated that ethanolic neem leaf extracts of less than 2000mg/kg of body weight administered orally did not result in mouse death. A study by Bandhopadhyay et al. 2004, showed the therapeutic potential of a lyophilized powder of an aqueous neem bark extract administered twice daily for 10 weeks at 30-60 mg dosages [12]. Neem is said to be more efficient compared to all other therapeutic plant extracts, inhibiting bacterial growth and biofilm-grown cells according to a study by Noor et al. in 2011 [13]. The antimicrobial activity of A. indica is thus established in a large number of previous studies [14 16]. Nimbolide, a component of neem revealed anti-cancer activity thereby giving way for further study on its anticancer effect [17]. On the other hand, Kurta or Swertia chirata has also shown a large number of medicinal properties. Kirata showed a high antimicrobial ability to combat both gram-positive and gram-negative microbes. Another study found that the ethanol extract of Swerchia chirata's leaves and stems had antibacterial activities and inhibited the growth of test organisms [6]. Muhammed et al. in study stated that various fractions of methanolic extract of S. chirata possessed antioxidant, antimicrobial, and brine shrimp cytotoxic and anti-leishmanial activities [18]. Maheshwaran et al. showed effective antimicrobial and cytotoxic activity of Neem when added with Stevia against gram-positive microorganisms when used as a mouthwash [19]. Herbal formulation of Pterocarpus santalinus with red sandal-mediated silver nanoparticles had a moderate cytotoxic effect against oral pathogens [20]. Silver nanoparticles synthesized with herbal formulation from Andrographis paniculata and Phyllanthus nitruri resulted in good activity against oral microorganisms [21]. Oral microbiota includes more than 700 species of bacteria and some fungi species. It exists primarily as biofilm, pellicle or coating on the tooth surfaces. These microorganisms primarily cause dental caries and other inflammatory diseases. Thus it is important for the formulation of new substances that prevent the growth and deposition of oral microbes. Thus Neem and Kirata when combined have certainly given beneficial effects such as superior antimicrobial and decreased cytotoxic effects.

## Limitations:

 This study was done under in vitro conditions. Hence the antimicrobial and cytotoxic activity of Neem and Kirata herbal formulation-mediated silver nanoparticle need to be evaluated clinically for their effectiveness and activity.

## Conclusion:

Neem and Kirata-induced silver nanoparticles are said to have antimicrobial and less cytotoxic properties in the research. It showed a strong inhibitory action while having few harmful side effects. To fully understand the advantages of the plants and the mechanisms underlying their actions, however, more research is necessary. This research thus offers a solid foundation for comprehending silver nanoparticles and their preceding characteristics of them. 
